# Coexistence via Resource Partitioning Fails to Generate an Increase in Community Function

**DOI:** 10.1371/journal.pone.0030081

**Published:** 2012-01-10

**Authors:** John P. DeLong, David A. Vasseur

**Affiliations:** Yale University, Department of Ecology and Evolutionary Biology, New Haven, Connecticut, United States of America; CNRS, University of Montpellier II, France

## Abstract

Classic ecological theory suggests that resource partitioning facilitates the coexistence of species by reducing inter-specific competition. A byproduct of this process is an increase in overall community function, because a greater spectrum of resources can be used. In contrast, coexistence facilitated by neutral mechanisms is not expected to increase function. We studied coexistence in laboratory microcosms of the bactivorous ciliates *Paramecium aurelia* and *Colpidium striatum* to understand the relationship between function and coexistence mechanism. We quantified population and community-level function (biomass and oxygen consumption), competitive interactions, and resource partitioning. The two ciliates partitioned their bacterial resource along a size axis, with the larger ciliate consuming larger bacteria than the smaller ciliate. Despite this, there was no gain in function at the community level for either biomass or oxygen consumption, and competitive effects were symmetrical within and between species. Because other potential coexistence mechanisms can be ruled out, it is likely that inter-specific interference competition diminished the expected gain in function generated by resource partitioning, leading to a system that appeared competitively neutral even when structured by niche partitioning. We also analyzed several previous studies where two species of protists coexisted and found that the two-species communities showed a broad range of biomass levels relative to the single-species states.

## Introduction

**Figure 1:**
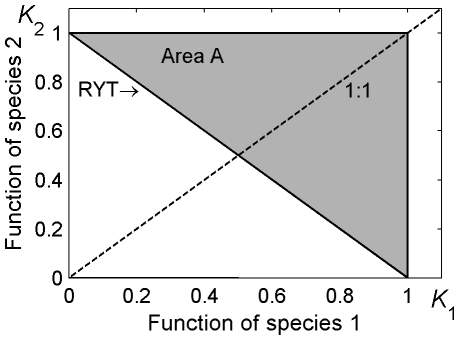
Conceptual figure showing the different positions of the steady-state of population density, biomass, or function under neutral or resource-partitioned dynamics. The carrying capacities, *K*, when alone of the two species are normalized at 1. Actual steady-states in a neutral system may drift along the RYT in either direction due to stochastic processes. Resource-partitioning increases total access to resources, causing the steady-state to rise above the RYT into Area A (gray triangle). Yet opposing forces could push the steady-state back down to the RYT, making the system appear neutral even though the underlying dynamics may not be.

The classic ecological explanation for coexistence is that species specialize on different parts of an available resource, leading to lower competition between species than within species [1]. Because of this specialization, the total resource uptake of species that coexist through this mechanism will be greater than the resource uptake of either species when they occur alone [2], [3]. This process is the basis of the biodiversity-ecosystem function hypothesis, where the “function” of a community is predicted to increase as additional species are added [4]–[8]. Function, in this sense, is any variable that reflects the ability of a population or community to use resources, such as the standing stock of biomass or the flux of energy [9], [10]. Drawn in state-space, the function of two species coexisting via a resource partitioning mechanism should occur above the line connecting the function of each species at steady-state when alone (the relative yield total, RYT; Fig. 1, after [11], [12]).

Alternatively, coexistence may occur when species are ecologically equivalent in terms of their competitive ability and niche [13], [14]. No increase in biomass or energetic flux is expected when neutral mechanisms generate coexistence, because there is no resource specialization and therefore no increase in resource uptake as species are added to the community [5], [12], [15]. Under Lotka-Volterra dynamics for two competing species, neutrality occurs when all competition coefficients are equal to unity, meaning that competitive effects are symmetrical within and across species. The steady-state of such a system in state-space resides along the RYT at a point that depends upon the carrying capacities of the two species. If competition coefficients are unity and carrying capacities are equal, the steady-state is located where the 1∶1 line of function between the species intersects with the RYT (Fig. 1). The steady-state in a neutral system may drift along the RYT but is not driven toward one end or the other by asymmetrical competitive effects.

Although there is considerable support for the hypothesis that community-level function increases with the diversity of the community, it is not always the case [9]. In some instances, total function may not increase as new species enter the community (generating a state below or along the RYT, Fig. 1). Perhaps this occurs because neutral mechanisms are enabling coexistence (meaning there is no force to increase function), or alternatively, other interactions may push the steady-state back down, such that the expected increase in function with coexistence is not observed [16]. It has been suggested that interspecific interference competition can have this type of countering effect [4], [17], [18], because interference competition causes a reduction in resource use independent of the resource levels [19].

We studied resource partitioning and community-level function of ciliates grown in laboratory microcosms. Ciliates often coexist in natural assemblages, where multiple species forage on a common resource such as bacteria [20]. Size-based partitioning of prey among protists of different sizes [21] and species [22] may contribute to coexistence of these organisms in natural environments, although competitive asymmetries are common and often lead to competitive exclusion in laboratory studies [23]–[25]. We obtained coexistence of *Paramecium aurelia* and *Colpidium striatum*, apparently due to resource partitioning of prey by size, but there was no increase in community-level function. We suggest these discrepant observations are most likely to be resolved by the presence of a negative feedback such as interspecific interference competition that counters the gains in function expected from resource partitioning. Finally, a survey of previous studies on protists indicates that coexistence is usually accompanied by either a decrease in function or by an increase in function much larger than expected for coexistence by resource partitioning mechanisms, and that neutral states are uncommon among these studies.

## Results

*Paramecium* is a slightly larger-bodied species than *Colpidium*. This difference was evident in these experiments, both in separate cultures and in two-species cultures. Overall mean volume of *Paramecium* individuals (n = 86) was 8.56×10^4^±2.96×10^3^ (SD) µm^3^, and for *Colpidium* individuals (n = 81) it was 5.04×10^4^±1.82×10^3^ µm^3^ (t = 10.04, df = 166, p = 0; using all measurements). Size differences between cells in the single-species and two-species cultures were not significant (*Paramecium*, t = −1.43, df = 84, p = 0.16; *Colpidium*, t = −0.67, df = 80, p = 0.5).

**Figure 2:**
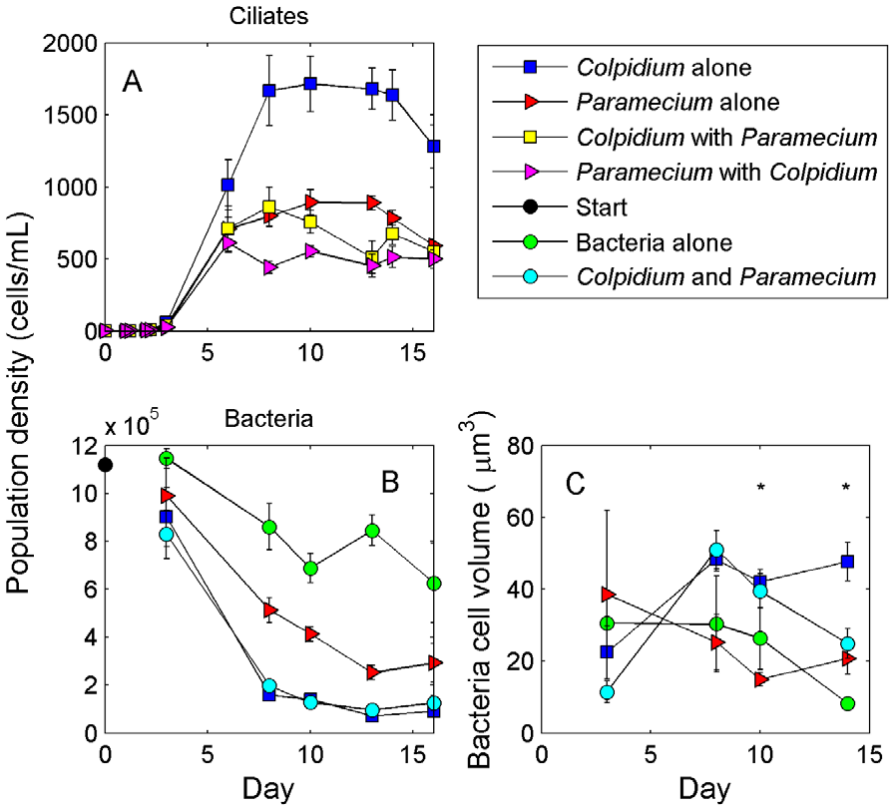
Population and size dynamics of protists and bacteria. (A) Population density of the *Colpidium striatum* and *Paramecium aurelia* in single and two-species communities and (B) density of the prey bacteria, *Bacillus subtilis*, in the different treatments. *Colpidium* and *Paramecium* coexisted in the two-species community, with *Colpidium* numerically dominant. *Colpidium* grazed *B. subtilis* to lower levels than *Paramecium*, but the overall population density of bacteria was similar between *Colpidium* and the two-species cultures. In (C), the estimated cell volume of *Bacillus subtilis* is shown by treatment, sampled four times during the course of the experiment (days 3, 8, 10, and 14). As time passed, a pattern of size-differentiation developed where the mean size of the bacteria increased in the *Colpidium* treatment and decreased in the *Paramecium* treatment. Differences between size of bacteria in the *Colpidium* and *Paramecium* treatments were nearly significant on day 8 and significant on days 10 and 14 (noted by asterisk). This indicates that *Colpidium* selected smaller bacteria than *Paramecium*, which is evidence of size-based resource partitioning between the two species.

Both *Paramecium* and *Colpidium* grew to steady-states (days 8–14) in their single-species cultures, and they coexisted in the two-species cultures through the course of the experiment with little evidence of a decline (Fig. 2A). *Colpidium* grew to higher densities (z = −10.4, P<0.001; linear mixed-effects model with treatment as a fixed effect and sampling day nested within replicate as a random factor; Fig. 2A) and biovolumes (z = −2.73, P = 0.03) than *Paramecium* in the single species cultures. In the two-species cultures, steady-states were similar between the species in terms of population density (z = −1.98, P = 0.20) and population biovolume (z = 2.13, P = 0.14).

When alone, *Colpidium* grazed bacterial populations to a lower level than *Paramecium* in terms of density (z = 6.12, P<0.001; Fig. 2B) but not in terms of biovolume (z = 0.82, P = 0.84). Bacteria levels in the cultures with both *Paramecium* and *Colpidium* were similar to cultures with only *Colpidium* (z = −0.28, P = 0.99; Fig. 2B) but all other contrasts were significant (z< = −0.91 or > = 5.9, P<0.001). With biovolume, bacteria alone cultures were all significantly different than other treatments (z<−3.6, P<0.002), but no other differences were detected between or among *Colpidium* and *Paramecium* cultures (|z|<1, P>0.85). Thus, there is little evidence for exploitation competitive asymmetries in the R* sense (R* is the quantity of resource unconsumed by a consumer at steady-state and is a measure of exploitation competitive ability [26]) between *Colpidium* and *Paramecium*, given the steady-state levels of bacteria biovolume.

Mean cell size of the bacteria populations in the single-species treatments diverged through the course of the experiment, so that by day 10 bacteria in the *Paramecium*-alone cultures were smaller than bacteria in the *Colpidium*-alone cultures (Day 3, F_3,17_ = 0.82, P = 0.51; Day 8, F_3,23_ = 2.38, P = 0.10; Day 10, F_3,23_ = 5.61, P = 0.006; Day 13, F_3,23_ = 16.67, P<0.001; Fig. 2C). Mean sizes of bacteria were usually in the 2–3 µm range, with the significant differences between mean size in the *Colpidium* and *Paramecium* treatments being about 1 µm. This indicates that the smaller *Colpidium* grazed more small bacteria, and the larger *Paramecium* grazed more large bacteria. This difference is evidence of resource partitioning among the two species and size-structuring of the bacteria populations by differential grazing pressure, although it took more than a week for these effects to be detectable.

**Figure 3:**
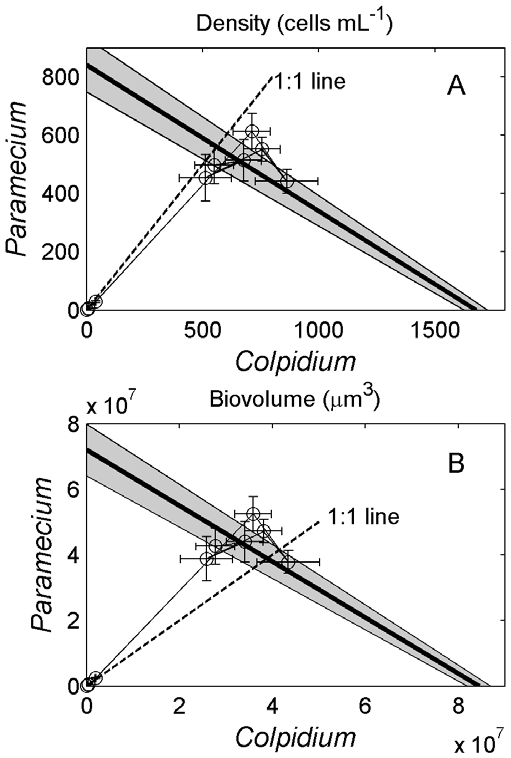
Population dynamics of *Colpidium striatum* and *Paramecium aurelia* in state-space. Plots show mean and standard errors of density (A) and biovolume (B). Both steady-states were centered along the RYT, which is a straight line connecting the steady-states of each species when alone. The gray shaded area along the trade-off perimeter is the 95% confidence intervals of the perimeter, as determined by the 95% CIs of the steady-states. Steady states that occur along this line when grown together indicate that the species are trading-off against each other in both density and biovolume, rather than gaining a boost in numbers of biovolume (over yielding) when grown together.

In state space, the two-species communities grew along the 1∶1 line and resolved to steady-states that straddled the RYT connecting the steady-states of either species alone (Fig. 3). Population density (Fig. 3A) and biomass (Fig. 3B) in the two-species cultures did not rise above the RYT as expected in a resource-partitioned system. In addition, whole community oxygen consumption did not differ among treatments (ANOVA, *F_2,17_* = 0.75, *P* = 0.49). When viewed on a numerical basis, *Colpidium* held a slight competitive advantage (points to the right of the 1∶1 line), and when viewed on a biomass basis, *Paramecium* held a slight advantage (points to the left of the 1∶1 line). *Paramecium* and *Colpidium*, however, were very similar in competitive ability given that the 95% confidence intervals of the LV competition coefficients for each species included one (Table 1).

**Table 1:** Steady-state biovolumes in single- and two-species cultures for *Paramecium* and *Colpidium*.

		K_c_	*Ĉ*	α_cp_
*Colpidium*	Mean (SE)	8.44×10^7^ (7.92×10^5^)	3.53×10^7^ (3.72×10^6^)	1.2
	95% low	8.59×10^7^	4.26×10^7^	0.9
	95% high	8.28×10^7^	2.81×10^7^	1.5

These values are used to estimate competition coefficients, and the standard errors of each are used to estimate 95% confidence intervals for each coefficient. *K* is carrying capacity and the α's are the Lotka-Volterra competition coefficients, subscripted *c* and *p* for *Colpidium* and *Paramecium*, respectively. Steady-state densities in two-species cultures are 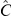 (*Colpidium*) and 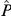 (*Paramecium*).

**Figure 4:**
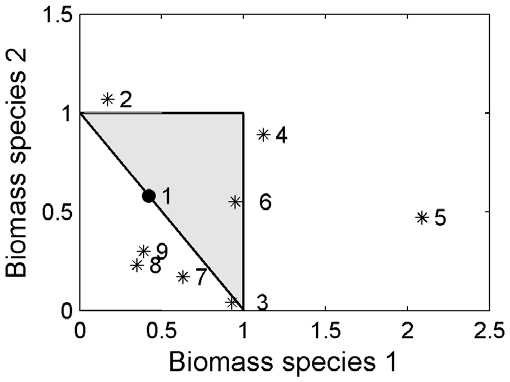
State-space for the steady-state population-level biomass from seven additional studies in the literature, in comparison with the results of this study. Three studies showed suppressed levels of function (#s 3, 7, and 8), one study showed elevated function consistent with classic resource-partitioning arguments (#6), and three studies showed increases in function for one species relative to its alone state, suggestive of a mutualism or other type of positive interaction (#s 2, 4, and 5). The studies were, with species 1 (on x-axis) listed first 1) *Colpidium striatum* versus *Paramecium aurelia* (this study, marked with solid circle), 2) *Blepharisma americana* versus *Paramecium tetraurelia* [38], 3) *Colpidium striatum* versus *Tetrahymena thermophyla* [38], 4) *Chilomonas paramecium* and *Colpidium striatum* (low nutrient levels; [37], 5) *Chilomonas paramecium* and *Colpidium striatum* (high nutrient levels; [37], 6) *Colpidium striatum* versus *Paramecium tetraurelia* (22°C; [22], 7) *Colpidium striatum* versus *Paramecium tetraurelia* (30°C; [22], and 8) *Paramecium aurelia* versus *Paramecium caudatum* [24].

We found seven additional cases of coexistence in protists (Fig. 4). These studies indicate that it may be common for some type of process to counteract the expected gains in function afforded to a system by resource partitioning, although ours was the only of these eight studies that showed direct evidence of resource partitioning. Three studies had coexistence steady states that suggest that countering effects of some sort were strong enough to suppress function below the RYT. Only one of the studies had a coexistence steady-state that occurred in the expected range for a competitive community where coexistence was maintained by resource partitioning (# 6 in gray triangle) and three studies had coexistence steady-states that suggest positive interactions, where at least one species fares better in coexistence than alone. This was mostly true for the two studies that included the flagellate *Chilomonas paramecium* (#s 4 and 5 in Fig. 4).

## Discussion

Our results suggest that the classical mechanism of resource partitioning was the basis of coexistence in this system. We observed size-based partitioning of the bacterial resource with the larger species (*Paramecium*) consuming on average larger bacteria than the smaller species (*Colpidium*, Fig. 2C), consistent with many previous observations [21], [27]. *Paramecium* and *Colpidium* also have been shown to partition bacterial prey by species or strain [22]. Such differential specialization on prey types should increase the spectrum of prey sizes that can be consumed, increasing the total amount of resource that can be utilized. Without a countering mechanism, classic theory predicts that we should have observed higher biomass and energetic fluxes in the two-species community relative to the single-species communities [3], [7], [11], [12].

Rather than the expected increase in function, however, the two-species steady-state in our experiment was characterized by biomass and oxygen consumption that was approximately equivalent to either species in their single-species cultures (Fig. 3). Furthermore, two-species communities did not consume more bacteria than single-species communities (Fig. 2B). This state is what we expect from a neutral system, where species are functionally redundant and demonstrate total resource use overlap. Given our documentation of resource partitioning, however, additional interspecific effects must be counteracting the gain in function [28]. It is unclear what these forces are, but theoretical studies have suggested that the expected gain in function resulting from resource partitioning could be dampened by interference competition [4], [17], [18], which is common among many small ectotherms including protists [19]. The effect of interference at the population level (regardless of the specific behavioral mechanism that generates it) is to reduce overall resource acquisition rates, potentially countering any gain in function generated by resource partitioning. Without direct estimates of interference competition, however, we cannot know whether this form of competition was the countering force. Alternatively, it is possible that resource partitioning along a size axis does not generate a large increase in function, and an actual increase went undetected. If this is the case, then the manner in which resources are partitioned may be important in terms of how much increase in function should be expected.

The steady-state in the two-species treatment was very consistent across replicates (Fig. 3). This consistency suggests that the steady-state was attractive, which lends further support to the interpretation that coexistence was generated by resource partitioning (ensuring weaker interspecific relative to intraspecific exploitative competition) and not neutral mechanisms. In contrast, stochasticity and drift should distribute the steady-states along the RYT when dynamics are governed by a neutral process. Although the steady state of the system is located on the RYT, the coalescence of replicates to the same location suggests instead that this position results from the opposing forces of resource portioning and some type of countering mechanism.

Previous experiments on coexistence in protists showed that coexistence steady-states may occur in a wide range of locations relative to the RYT (Fig. 4). Of the eight experiments that we found in the literature, including this one, the most common outcome (four of the studies) was that the biovolume of the community was not greater in coexistence than in the single-species cultures, which again could indicate that a mechanism other than resource partitioning is enabling coexistence. Ours is the only experiment whose function levels are consistent with neutral mechanisms, although others have suggested that neutral mechanisms are involved in coexistence in natural assemblages of protists [20]. It is interesting to note that in three of the eight studies there is evidence of a possible mutualism between the species, as some species function at higher levels in coexistence than they did when alone [29]. This was particularly true for experiments using the flagellate *Chilomonas paramecium*, which may thrive on bacteria or organic compounds in the media [30], suggesting that function (biomass in these studies) is altered by a mutualistic interaction [4].

It is interesting that the different measures of competitive interactions did not all agree. In this experiment, *Colpidium* had a lower R* than *Paramecium* when considering bacteria numbers, but not on a biovolume basis. Thus, one could conclude that either the two ciliate species actually showed symmetrical exploitation competitiveness or that *Colpidium* had a slight competitive advantage over *Paramecium*, depending on the metric used. In contrast, Lotka-Volterra competition coefficients suggested that *Paramecium* and *Colpidium* were competitively very similar (Table 1). We suggest that some of these disparities might arise because the Lotka-Volterra coefficients actually measure the combined effects of exploitation and interference effects, and thus as measures of exploitation competition resulting from resource specialization, they are overestimates. This is because the observed coefficients are generated by the effects of both exploitation and interference competition, but the Lotka-Volterra model does not explicitly include terms for interference competition. If the coefficients could somehow be corrected for interference, we suggest that they would both be <1, indicating resource partitioning in the classical sense, and resolving the mismatch between the expectation from resource partitioning and the appearance of functional redundancy. We encourage additional work that will resolve the differences among different measures of competition and how to assess the effects of interference on competition coefficients and function.

Niche-partitioning along a size axis has been found for a variety of organisms [31], [32] and size-based niches are generally thought to be important for food-web structure [27], [33], [34]. In this study, the prey was size-partitioned between *Colpidium* and *Paramecium* without also being partitioned by species. This indicates that size-partitioning of a resource along a body size axis can facilitate coexistence, but it may alter prey dynamics when different-sized prey are of different ages. Heavy grazing pressure on small (young) cells may reduce the number of large cells that are produced, and heavy grazing on large (old) cells may reduce the rate of new cell production. In this study, our replenishment of the prey population with naïve, ungrazed prey every 2–3 days maintained an influx of prey that covered the normal range of body sizes of *B. subtilis*, potentially limiting this effect. Nonetheless, such prey impedance may represent an alternative mechanism by which function may be depressed below that expected from niche-partitioning. In addition, it is possible that bacterial densities may have influenced size via density-dependent affects on resource levels, but further work will be needed to evaluate this possibility. Future work should aim to address how dynamic feedback among resource size classes alters coexistence and community properties.

## Methods

### Experimental set-up

We acquired the bactivorous ciliates *C. striatum* (hereafter referred to as *Colpidium*) and *P. aurelia* (hereafter *Paramecium*, which is some member of the *P. aurelia* complex with an unclear specific identity) from Carolina Biological Supply (Burlington, NC, USA). Individual ciliates of both species were isolated, repeatedly washed with sterile media, and maintained in stocks inoculated with *B. subtilis*. Ciliate populations were allowed to grow for many generations on *B. subtilis* prior to the initiation of the experiment.

We grew replicate populations of *Colpidium* and *Paramecium* to steady-states in both single-species and two-species microcosms and set up replicate microcosms of *B. subtilis* without ciliates (six microcosms for *Colpidium* alone, six for *Paramecium* alone, six for both species together, and six with bacteria only). We used 50 mm diameter Petri dishes containing 5 mL of media as microcosms, maintained at 21°C in an incubator. About 200 mL of autoclaved media (liquid protozoan concentrate diluted 1∶20 in Spring Water, both from Carolina Biological Supply) was inoculated directly from plated *B. subtilis*. After two days the media was filtered through a 70 µm cell-strainer (to remove bacterial flocs) and 5 mL of this bacterized media was added to each microcosm. Initial bacterial density and biovolume were determined with the filtered stock (see below). Then, we extracted protists from stock cultures, washed them in sterile media, and transferred them to the microcosms, watching through a microscope to ensure that all individuals were transferred. Five individuals went into the single-species treatments, and 10 individuals (five of each species) went into the two-species treatments. We also filled one additional microcosm with 5 mL of media to serve as an evaporation control. We weighed this control microcosm on the first day and on every subsequent weekday, to determine the amount of evaporative water loss. Each weekday, prior to any sampling or counting, all microcosms were topped off with micro-filtered water (Barnstead Nanopure Diamond system) in the amount of the evaporated loss.

Every Monday, Wednesday, and Friday, 0.2 mL of culture was extracted from each microcosm, and 0.2 mL of fresh, bacterized, 70-µm-strained media was added back. Fresh media was the same as the initial stock, bacterized two days prior (three days in the case of weekends) with plated *B. subtilis*. Replenishment of bacteria helped to ensure that the bacteria was dominated by *B. subtilis*, to minimize successional changes in the bacteria due to grazing, and to minimize evolutionary changes in the bacteria that might alter prey palatability [35]. This technique ensured that function changes were not due to changes in traits of the prey but were rather due to the resource partitioning mechanism in which we are interested. The experiment was run for 15 days, which is long enough for competitive asymmetries to arise in rigorously maintained microcosms [24].

### Determination of numbers and biovolumes

We enumerated bacteria with a laser particle counter (Spectrex PC-2200, Redwood City, CA). For each measurement, we took 0.1 mL of culture, diluted into a 100 mL micro-filtered water blank, and determined the number of particles per mL for each size category of 1 to 10 µm Estimated Spherical Diameter (ESD). We sampled each 100 mL blank prior to adding the sample to adjust for background particles. Very few background particles were detected; most blanks had no particles above 1 µm. We took four measures of the sample and used the mean size-frequency distribution per replicate as our estimate. We calculated the total biovolume of bacteria by multiplying the density at each size by the volume for each diameter, assuming cells are spheres. Although *Bacillus* species are rod-shaped, the particle counter gives dimensions as ESD, and so this method is appropriate.

We counted protists manually through a dissecting stereomicroscope (Leica M165C). We used a density-scaled procedure. For low densities (up to about 10 cells mL^−1^), we counted the entire protist population in the dish, assisted with a clear, gridded plate placed below the Petri dish. For medium densities (about 10–50 cells mL^−1^), we counted the protists in the 0.2 mL of media extracted from the culture. And at higher densities (>50 cells mL^−1^), we counted protists in just 0.1 mL of the extracted 0.2 mL. Thus, our counting regime enabled us to tune the counting to minimize sampling errors while maintaining a consistent extraction and replenishment protocol through the course of the experiment.

At the steady-states, we measured the body size of both protist species. *Colpidium* and *Paramecium* cells were extracted from a mixed sample from the single-species and two-species microcosms and photographed using a digital camera (Leica DFC420 attached to the counting microscope). Lengths and widths were measured with the cross-bar tool in the Leica Application Suite, and cell volume was calculated for each cell using the formula for a prolate spheroid.

### Measuring competition

We estimated Lotka-Volterra competition coefficients (α's, subscripted *c* for *Colpidium* and *p* for *Paramecium*) from the steady-state solutions of the Lotka-Volterra model solved for the respective species [36]:
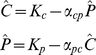
(1)We used the mean steady-state biovolumes of each species in monoculture for the *K*'s and in polyculture for the equilibrium coexistence densities (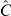 and 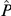, for *Colpidium* and *Paramecium*, respectively). It is important to note that these competition coefficients combine the effects of both exploitation (niche difference) and interference competition into one parameter.

### Determination of energetic fluxes

We measured oxygen consumption of the microcosm communities with a fluorescent oxygen probe (DO-400, Golden Scientific, Temecula, CA). Measurements were made one time for each microcosm (giving 18 measurements, as we did not measure the bacteria-alone cultures). At steady-state, beginning on day 10 when clear steady-states and resource partitioning was were achieved, we extracted 0.3–0.4 mL of the microcosm with a 1 mL graduated syringe without needle tip, and inserted the probe into the open end of the syringe. We expunged all air from the syringe chamber with the plunger and sealed the tip of the syringe with the probe inside using tacky rubber. The entire system was kept in an incubator to maintain stable temperature and pressure.

### Comparison with data from the literature

Finally, we examined previous studies on coexistence of protists to compare with our results. We searched for studies in online databases using keywords that included various protist species, authors, and biological terms such as coexistence. We searched in the literature cited for additional work. We included only studies where presented time series showed stable coexistence of two species and where single-species steady-state data also were available. Although many competition studies have been conducted with protists, relatively few have produced coexistence, and not all of these provided time-series data that could be used here. Most competitive trials with protists ultimately end in the extinction of one species, so despite the vast literature using these organisms, there are surprisingly few examples with which to compare. In order to compare the outcomes of the different experiments quantitatively, we normalized the population-level biomasses when alone as 1, and calculated the relative biomass when coexisting with another species as biomass in coexistence/biomass when alone. Then, we plotted all of the outcomes in one state-space figure. Population-level biomasses were taken either directly from the original sources [22], [37], [38], or by digitizing time-series data from the original source and taking the mean of the final five measurements and multiplying by the average body mass [24].
